# The Antagonistic Influence of Phytic Acid on Zinc Absorption: An *In Vitro* Comparison of Inorganic and Chelated Trace Mineral Sources

**DOI:** 10.3390/nu18010046

**Published:** 2025-12-22

**Authors:** Niamh Rock, Martin Clynes, Karina Horgan, Richard Murphy, Finbarr O’Sullivan, Joanne Keenan

**Affiliations:** 1 Life Sciences Institute, Dublin City University, D09 Y074 Dublin, Ireland; martin.clynes@dcu.ie (M.C.); finbarr.osullivan@dcu.ie (F.O.); 2 School of Biotechnology, Dublin City University, D09 Y074 Dublin, Ireland; 3 SSPC The Research Ireland Centre for Pharmaceuticals, University of Limerick, V94 T9PX Limerick, Ireland; 4 Alltech Bioscience Centre, Summerhill Road, Sarney, A86 X006 Dunboyne, Meath, Ireland; khorgan@alltech.com (K.H.); rmurphy@alltech.com (R.M.)

**Keywords:** zinc, phytic acid, bioaccessibility, bioavailability, bisglycinate, proteinate, trace mineral chelate

## Abstract

**Background/Objectives:** Zinc, an important trace metal, requires daily intake but dietary antagonists including phytic acid reduce its absorption. It is unclear if phytic acid affects zinc absorption at the level of bioaccessibility (how much soluble zinc is available from digestion) or bioavailability (how much zinc is absorbed by the intestine). This study investigates at which level this occurs at and if the zinc source alters the response. **Methods:** Following a standardised *in vitro* digestion (INFOGEST), the yield of soluble zinc was measured as the bioaccessible fraction from inorganic and chelated zinc sources, with and without phytic acid. Bioavailability was assessed by measuring cellular zinc uptake in intestinal cell lines (Caco-2 and IPEC-J2). **Results:** Phytic acid affected the bioaccessibility of zinc, with varying impacts depending on the zinc source. Zinc proteinate had the highest bioaccessibility (42%) without phytic acid, while inorganic zinc sulphate (24%) and zinc bisglycinate (27%) were lower. ZnSO_4_ was more susceptible to phytic acid antagonism than chelated zinc sources (from 2:100 molar ratio of phytic acid: zinc), while the chelated zinc sources were only affected at a molar ratio of 4:100, with zinc bisglycinate being more susceptible than zinc proteinate. Cellular zinc uptake (bioavailability) and toxicity at equimolar concentrations were unaffected by phytic acid. **Conclusions:** This study found that phytic acid affected bioaccessibility, not bioavailability. The zinc source impacts the response. Zinc proteinate was consistently more bioaccessible while both chelated zinc sources were less susceptible to phytic acid than inorganic zinc.

## 1. Introduction

Zinc is an important micronutrient that plays a role in cell signalling, growth, differentiation, and immunity. Zinc deficiency causes growth failure or retardation, hair loss, diarrhoea, and thickening and hyperkeratinisation of epidermis [1]. While on a cellular level, zinc deficiency has adverse effects on a variety of processes such as cell-mediated innate immunity and natural killer cell activities. Despite its importance, zinc cannot be stored in the body and must be nutritionally sourced each day [2]. The recommended daily allowance (RDA) of zinc intake is 8–11 mg/day for adult humans [3] with higher levels for farm animals, ranging from 40 to 110 Mg/kg for broiler chickens [4] to 150 mg/kg for newly weaned piglets [5]. Regardless of the RDA, zinc ingested does not equate to zinc available for absorption [6].

A major obstacle in zinc uptake is the presence of dietary antagonists including phytic acid, iron, copper, cadmium, and calcium [7,8]. The most significant antagonist is phytic acid [9], which reduces the bioavailability of divalent metals such as zinc *in vivo* through the formation of stable complexes at acidic pHs, i.e., during the gastric phase of digestion [10]. The formation of these phytic acid–mineral complexes is due to the strong chelating ability of the six reactive phosphate groups resulting in the reduced availability of zinc for absorption by enterocytes [11]. These complexes are transported to the intestine where the neutral pH causes them to become insoluble *in vivo* [12,13]. Monogastric animals such as humans and pigs lack phytase enzymes in the gastrointestinal tract required to adequately degrade phytic acid [14], resulting in a significant decrease in bioavailability of dietary phosphorus and micronutrients such as zinc [15]. This is problematic for those on plant-based diets which are rich in phytic acid [16,17], particularly people in third-world countries, vegetarians, vegans, and the elderly [18,19]. In addition, it presents an agricultural and an environmental challenge for livestock as unabsorbed minerals are excreted as environmental pollutants [20].

To fight deficiency in humans and increase health and productivity in agricultural and livestock settings, two approaches are used. The first is the use of chelated or organic sources of bio-metals such as copper, zinc, iron, manganese, and selenium, which have been found to offer increased bioavailability [4,21,22,23]. A variety of chelating agents are used including amino acids [24], bisglycinate [25], gluconate [26], and proteinate [27].

Secondly, phytase is often used as a supplement in monogastric diets, whereby phytic acid breakdown increases the bioaccessibility of zinc, thereby reducing the symptoms of ‘leaky gut’ and diarrhoea [28,29]. Phytases dephosphorylate phytic acid, resulting in the release of bound cations and phosphorus from phytic acid, thus increasing the digestibility of proteins, sugars, and fats [20,30]. Furthermore, it was noted that the feed conversion ratio and body weight were significantly greater in animals receiving a diet with the recommended level of phosphorus or a low-phosphorus diet supplemented with phytase compared to animals with a low-phosphorus diet only [31].

In most *in vivo* systems, only bioavailability is reported, as the bioavailability of zinc cannot be readily separated from its bioaccessibility *in vivo*. Consequently, the terms are often used interchangeably; however, bioaccessibility describes how much of a (micro) nutrient, e.g., zinc, is available for absorption following digestion, whereas bioavailability refers to how much of a (micro) nutrient, e.g., zinc, is absorbed by the small intestine [32].

The utilisation of a standardised static *in vitro* digestion (INFOGEST) for metal compounds, such as those containing zinc, presents the opportunity to dissect the contribution of antagonists on bioaccessibility and bioavailability [33,34]. This study aimed to investigate the effect of the zinc source (i.e., inorganic and chelated forms) and the antagonistic impact of phytic acid on bioaccessibility using the standardised static *in vitro* digestion on bioavailability in the intestinal cell line models, Caco-2 and IPEC-J2.

## 2. Materials and Methods

### 2.1. Materials

IPEC-J2 (Cat. ACC701, DSMZ) and Caco-2 (Cat. HTB-37, ATCC) cells were grown in DMEM (Cat. 61965059) supplemented with 1% HEPES (Cat. H4034), 1% sodium pyruvate (Cat. 11360039), and 10% heat-inactivated FCS (Cat. 10270-106). Both cell lines were tested routinely for mycoplasma and found to be negative. All media and serum were obtained from Thermo Fisher Scientific (Dublin, Ireland). Phytic acid (Cat. P8810) and phytase from wheat (Cat. P1259) were obtained from Merck (Wicklow, Ireland). *Aspergillus niger* phytase was supplied by Natuphor. The composition of organic chelated zincs can range from simple amino acid chelates to more complex hydrolysates and proteinates. Thus, a Zn bisglycinate was chosen as an amino acid chelate. Zn proteinate formed from soy-derived amino acids and peptides was chosen as a more complex chelate to be compared to inorganic ZnSO_4_. Both chelated zinc sources were obtained from independent distributors.

### 2.2. In Vitro Digestion

A standardised static *in vitro* digestion protocol described by Brodkorb et al. [33] was employed. All zinc sources were applied at equimolar concentrations prior to digestion. The zinc was solubilised in simulatory salivary fluid (SSF) at pH 7. Phytic acid and phytase were added in the oral phase of digestion, for relevant samples. The solutions were mixed in an incubator at 37 °C for five minutes. A digested control, which consisted of all components of the *in vitro* digestion except for zinc, was included to account for components in the digestion process.

In the gastric phase, simulatory gastric fluid (SGF) was added and the pH was adjusted to pH 3, prior to the addition of 2000 U/mL pepsin (Cat. P6887, Merck) and incubation at 37 °C for two hours. Intestinal phase pH was adjusted to pH 7. Simulatory intestinal fluid (SIF), pancreatin (Cat. P1750, Merck), and bile salts (Cat. B8631, Merck) were added to a final concentration of 100 U/mL and 10 mM, respectively. The concentration of bile salts was determined using a bile assay kit (Cat. MAK309, Merck) and a trypsin assay kit (Cat. Ab102531, Abcam, Amsterdam, The Netherlands) was used to determine the tryptic activity of the pancreatin.

The solutions were incubated at 37 °C for a further two hours. The insoluble fraction was removed from the digested solutions through multi-step centrifugation (3330 g for 10 min at 4 °C retaining supernatant and 8817 g for 15 min at 4 °C). The digested solutions were then centrifugated through 10 kDa filters (Cat. UFC 901024, Merck, Millipore, and a 0.2 μm filter was used to filter-sterilise the solutions prior to storage at −80 °C.

Soluble zinc was measured using a colourimetric assay as described by the manufacturer (Cat. MAK-032, Merck; Cat. MET-5138, CliniSciences, Dublin, Ireland). An acid precipitation using the deproteinising solution provided with the kit was performed on soluble zinc digests and digests were further diluted with deionized water prior to analysis. The colourimetric zinc assay was validated using flame atomic absorption spectrometry (FAAS) (Supplementary Figure S1). The bioaccessibility or yield was calculated as a percentage of the zinc in the soluble fraction compared to the starting zinc concentration prior to digestion.

### 2.3. Detection of Phytic Acid and Confirmation of Degradation by Phytase

The presence of phytic acid was detected by running samples or digests on 33% polyacrylamide gels and staining with 0.5% toluidine blue [35]. Specifically, phytic acid concentrations of 0.5–20 nanomoles (reflecting the levels used in the digestion) were heated at 37 °C or 55 °C for one hour both with and without phytase. Samples were centrifuged at 15,800 g for 5 min and Orange G dye (Cat. R0631, Thermo Fisher Scientific) was added and mixed. A volume of 5 μL of O’RangeRuler 10 bp DNA Ladder (Cat. SM1313, Thermo Fisher Scientific) was loaded as a molecular weight marker and 20 μL of each sample was loaded onto 33% acrylamide gels and it was run at 170 V for four hours. Gels were stained with 0.5% toluidine blue (0.5% toluidine blue, 25% methanol, 12% glycerol) for 15 min, destained (25% methanol, 5% glycerol, 70% UHP H_2_O) for a further 15 min, and scanned using an EPSON (Suwa, Japan) scanner. Densitometry was performed using the CLIQS software (version 1.6.454).

### 2.4. Zinc Toxicity Assays

Proliferating IPEC-J2 and Caco-2 cells were exposed to 100 μM of digested zinc with or without phytic acid or digested controls (without zinc) for 72 h at 37 °C with 5% CO_2_. Acid phosphatase activity was measured using *p*-nitrophenyl phosphate (*p*-NPP) to determine zinc toxicity as the end point of the assay.

### 2.5. Zinc Bioavailability Assays

Zinc bioavailability was determined by measuring the level of uptake in intestinal cells. Proliferating IPEC-J2 and Caco-2 cells were seeded at 4 × 10^5^ cells and 7 × 10^5^ cells per 25 cm^2^, respectively, to be 80% confluent after overnight incubation at 37 °C. Cells were exposed to phosphate-free Hank’s buffered saline solution (pf-HBSS) (1.26 mM CaCl_2_, 1.1 mM MgCl_2_, 5.36 mM KCl, 137 mM NaCl, 5.5 mM Glucose, 30 mM HEPES, pH 7.4) for 20 min at 37 °C to remove zinc present in serum-supplemented medium and reduce phosphates which interfere with the zinc assay kit. Cells were then exposed to 100 μM of digested zinc or digested controls for two hours, reflecting the average time for zinc exposure in the small intestine *in vivo*. Pre-warmed pf-HBSS was used to gently wash the cells three times and cells were lysed using 200 µL of lysis buffer (7 M Urea, 2 M Thiourea, 4% CHAPS, 30 mM Tris). The protein concentration of each lysate was determined (after acidifying samples with 0.12 M HCl) using a Bradford assay (Cat. 5000006, Accuscience, Kildare, Ireland). Zinc levels in the lysates were determined using a colourimetric zinc assay (Cat. MAK-032, Merck: Cat. MET-5138, Clinisciences) following deproteinization as described by the manufacturer and expressed in nanomoles per milligram of cell protein. The zinc concentration of the lysates was above the lower limits of detection (0.78 µM) and quantification (2.37 µM) for the colourimetric assay and displayed negligible interference from copper, iron, or the 2D lysis buffer (Supplementary Figure S1D,E). These concentrations were confirmed using flame atomic absorption spectrometry (FAAS) (Supplementary Figure S1F,G).

### 2.6. Statistical Analysis

Student’s *t*-test with unequal variance and a two-tailed distribution was used to determine statistical significance between two conditions, e.g., ZnSO_4_ and Zn proteinate. To compare all three zincs across all groups, a two-way ANOVA was used. It was assumed that the data within each group were normally distributed, the variances of each group were equal, and the observations were independent of each other. Subsequently, Tukey’s multiple comparisons test was performed to compare values across a condition, e.g., one phytic acid/zinc molar ratio for the three zincs. A *p*-value of <0.05 was considered statistically significant.

## 3. Results

### 3.1. Differences in Inorganic and Organic Zinc Bioaccessibility and Impact of Phytic Acid

To assess the bioaccessibility of zinc from inorganic and chelated sources, as well as the impact of phytic acid, zinc and zinc/phytic acid mixes were subjected to a standardised *in vitro* digestion [33]. Bioaccessible zinc was measured as the zinc recovered in the soluble fraction of the digestion. The World Health Organisation (WHO) stated that less than 15% of zinc is absorbed from complex foodstuff when the molar ratio of phytic acid/zinc is greater than 15:1 [36]. However, without additional foodstuff, in the standardised in vitro digestion model, even a ratio of 2:1 phytic acid/zinc resulted in complete loss of zinc in the soluble fraction of the digests. Consequently, molar ratios of 0:100, 1:100, 2:100, 4:100, and 10:100 phytic acid/zinc were chosen for digestion, keeping the starting level at 200 nanomoles of zinc.

**Figure 1:**
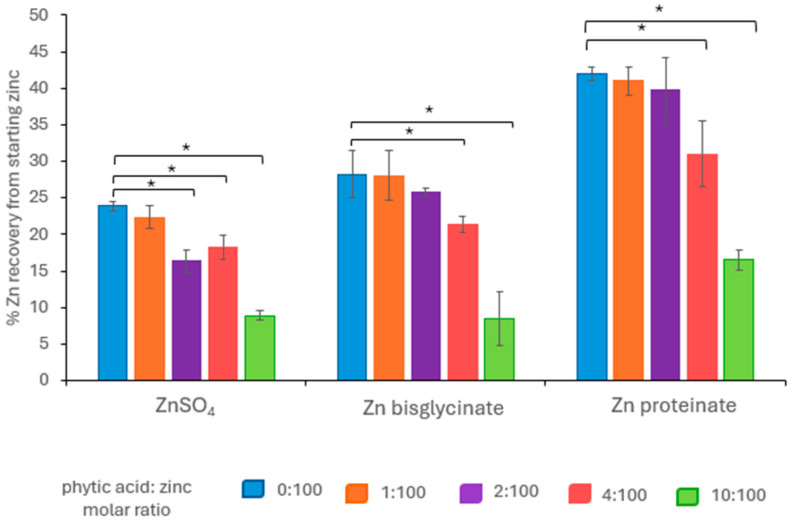
Bioaccessibility of various zinc sources from starting zinc following *in vitro* digestion in the presence of phytic acid at a variety of phytic acid: zinc molar ratios; 0:100 (blue); 1:100 (orange); 2:100 (purple); 4:100 (pink); 10:100 (green) (*n* = 5). * Represents a significant difference between the two data points indicated where *p* ≤ 0.05 and is detailed in Supplementary Tables S1–S4.

Prior to the addition of phytic acid, the organic zinc sources were found to be more bioavailable than inorganic ZnSO_4_ (Figure 1). Zinc proteinate (Zn proteinate) had the highest bioaccessibility at 42% and was 1.8-fold higher than ZnSO_4_ (*p* < 0.01) at 24%. Zn proteinate recovery was also 1.5-fold higher than zinc bisglycinate (Zn bisglycinate) (*p* < 0.01) which had a 28% recovery. Zn bisglycinate recovery was not statistically different to that of ZnSO_4_.

When phytic acid was included in the digestion, there were further reductions in zinc recovery with increasing levels of phytic acid beyond the molar ratio of 1:100 (Figure 1). The zinc sources, however, responded differently in their interaction with phytic acid; the chelated zinc sources were less impacted by phytic acid than inorganic zinc. ZnSO_4_ bioaccessibility was significantly reduced at a phytic acid/zinc molar ratio of 2:100 (*p* < 0.01) (Tables S1A and S3) and was significantly lower than Zn bisglycinate at the same molar ratio of 2:100 (*p* < 0.01) (Tables S1B and S4).

In comparison, Zn proteinate had significantly higher recoveries at all molar ratios compared to both ZnSO_4_ and Zn bisglycinate (Tables S1B and S4), with a recovery of 16% at the 10:100 phytic acid/zinc, in comparison to 8% for both ZnSO_4_ and Zn bisglycinate (*p* < 0.01) (Figure 1). This indicated that Zn proteinate was the most bioaccessible of the three zinc sources tested, even at the highest molar ratio tested for phytic acid/zinc of 10:100.

### 3.2. Phytase Ameliorates the Negative Impact of Phytic Acid on Zinc Bioaccessibility

As phytic acid reduced the bioaccessibility of zinc sources, the inclusion of phytase in the digestion (to break down the phytic acid) should restore the zinc bioaccessibility. The activity of the two phytase sources was investigated (*Aspergillus niger* and wheat phytase) initially, followed by their impact on the recovery of soluble zinc on inclusion during digestion. Polyphosphates like phytic acid can be detected using high-percentage acrylamide gels stained with toluidine blue [35]. To confirm the detection of phytic acid, a range of concentrations were tested to reflect the levels of phytic acid used in the digestions. The concentrations of 0.5–20 nmoles of phytic acid cover the phytic acid: zinc ratios 1:100 (2 nmoles) to 10:100 (20 nmoles) were used in the digestion. Both 37 °C and 55 °C incubation temperatures were selected, as 37 °C is both biologically relevant and the optimum temperature for *Aspergillus niger* phytase activity, whereas 55 °C was the optimum temperature for wheat phytase activity.

#### 3.2.1. Phytase Breaks Down Phytic Acid at 37 °C and 55 °C

**Figure 2:**
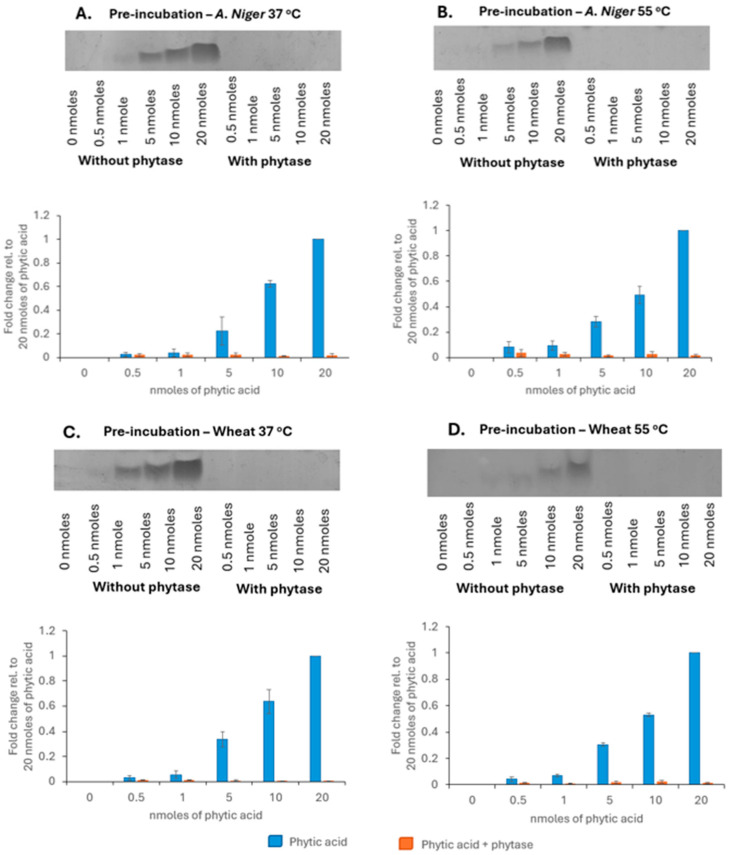
PAGE analysis showing effect of treatment of phytic acid standards with (**A**) phytase from *Aspergillus niger* at 37 °C, (**B**) phytase from *Aspergillus niger* at 55 °C, (**C**) phytase from wheat at 37 °C, and (**D**) phytase from wheat at 55 °C. Representative blots shown (*n* = 3).

Phytic acid was detected on the gels and the band intensity increased with increasing phytic acid concentration in a linear fashion from 1 nmoles (Figure 2). There were no bands detected in the phytic acid samples treated with *Aspergillus niger* phytase at 37 °C (Figure 2A) or at 55 °C (Figure 2B), indicating phytic acid breakdown. Wheat phytase likewise degraded phytic acid at both 37 °C (Figure 2C) and 55 °C (Figure 2D). These results suggested that both phytases could be used at 37 °C, which is the temperature for the digestions. Finally, to investigate if any phytic acid remained in the soluble fractions after digestion, samples of digests were run on 33% gels and stained with toluidine blue.

**Figure 3:**
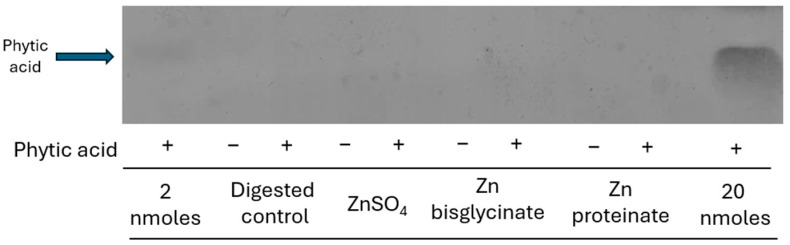
PAGE analysis of zinc sources digested *in vitro* with (+) or without phytic acid (−) in a molar ratio of 10:100 phytic acid/zinc. Representative blot shown (*n* = 3).

No phytic acid bands were detected post-digestion in the soluble fraction, supporting the observation that the zinc and phytic acid had formed insoluble complexes [10] (Figure 3).

#### 3.2.2. *Aspergillus niger* Phytase Ameliorates Phytic Acid: Impact on Bioaccessibility

**Figure 4:**
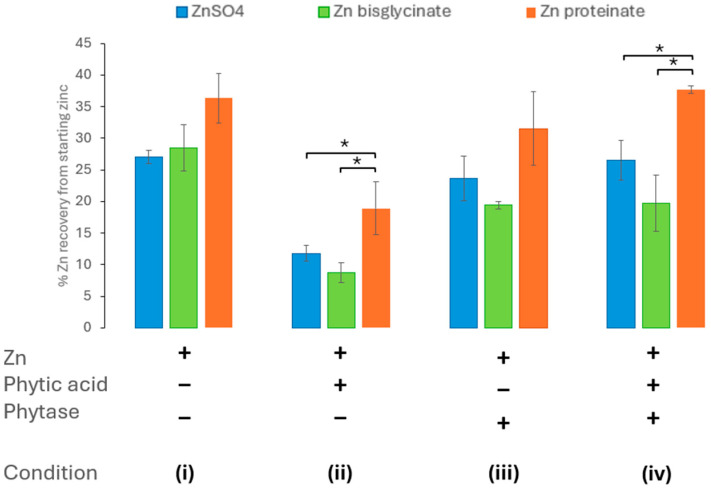
Effect of phytase (*Aspergillus niger*) activity on zinc bioaccessibility (*n* = 3) with (**i**) no added phytic acid or *Aspergillus niger* phytase, (**ii**) phytic acid in a 10:100 phytic acid/zinc molar ratio, (**iii**) added *Aspergillus niger* phytase, (**iv**) phytic acid in a 10:100 phytic acid/zinc molar ratio, and *Aspergillus niger* phytase added. * represents a significant difference between the two data points indicated where *p* ≤ 0.05 and is detailed in Supplementary Tables S5–S8.

To assess the impact of including *Aspergillus niger* phytase on zinc bioaccessibility, it was introduced in the oral phase of *in vitro* digestion at the same time as zinc and phytic acid. This was to accurately mimic digestion *in vivo*. The inclusion of *Aspergillus niger* phytase in the *in vitro* digestion resulted in the amelioration of the effect of phytic acid (at molar ratio of 10:100 phytic acid/zinc) on the bioaccessibility of the ZnSO_4_ and Zn proteinate (Figure 4, condition iv). Furthermore, when included in isolation, i.e., without the addition of phytic acid, the *Aspergillus niger* phytase itself did not impact the zinc recovered following the *in vitro* digestion (Figure 4, condition iii). The chelated Zn proteinate was the most bioaccessible following *in vitro* digestion, even in the presence of phytic acid and *Aspergillus niger* phytase (Figure 4, condition ii). It was evident that the recoveries between the two chelated zinc sources, Zn bisglycinate and Zn proteinate, were significantly different under all conditions i–iv (*p* < 0.01) (Supplementary Tables S5B and S8).

#### 3.2.3. Wheat Phytase Does Not Ameliorate Phytic Acid: Bioaccessibility Impact

**Figure 5:**
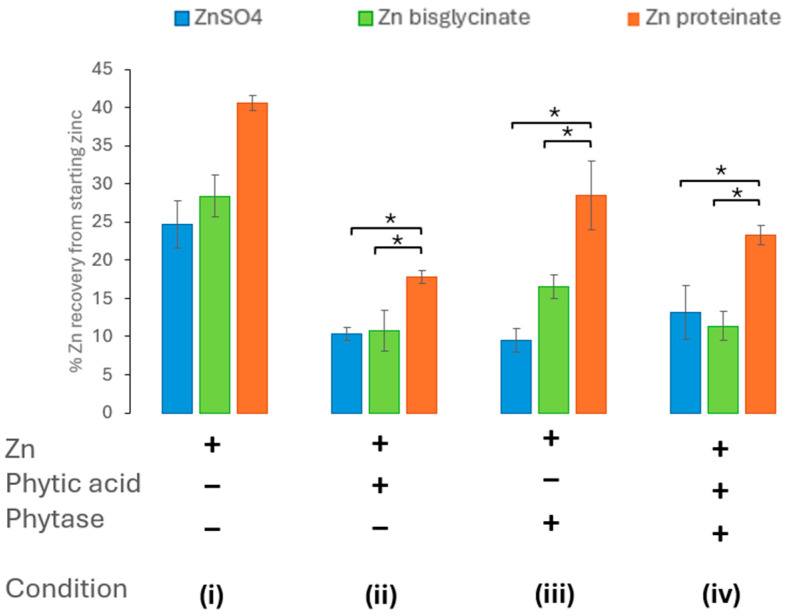
Effect of phytase (wheat) activity on zinc bioaccessibility (*n* = 3) with (**i**) no added phytic acid or wheat phytase, (**ii**) phytic acid in a 10:100 phytic acid/zinc molar ratio, (**iii**) added wheat phytase, (**iv**) phytic acid in a 10:100 phytic acid/zinc molar ratio, and wheat phytase added. * represents a significant difference between the two data points indicated where *p* ≤ 0.05 and is detailed in Supplementary Tables S9–S12.

When wheat phytase was introduced in the oral phase of the digestion, no amelioration of the impact of phytic acid was seen, regardless of the zinc source (Figure 5). In fact, the wheat phytase on its own reduced the recovery of zinc and there was no significant benefit in incorporating the phytase into the digestion (Figure 5). The bioaccessibility of inorganic zinc, ZnSO_4_ (*p* < 0.01), and the zinc chelates, Zn bisglycinate (*p* < 0.01) and Zn proteinate (*p* < 0.05), was significantly impacted by the inclusion of wheat phytase (Supplementary Tables S5A and S6). There was also a significant difference between the two zinc chelates when digested *in vitro* with wheat phytase (*p* < 0.05) (Supplementary Tables S9B and S12). The inclusion of both phytic acid at molar ratio of 10:100 (phytic acid/zinc) and wheat phytase in the *in vitro* digestion did not restore the bioaccessibility of any zinc source (Figure 5).

### 3.3. Impact of Phytic Acid on In Vitro Zinc Bioavailability

**Figure 6:**
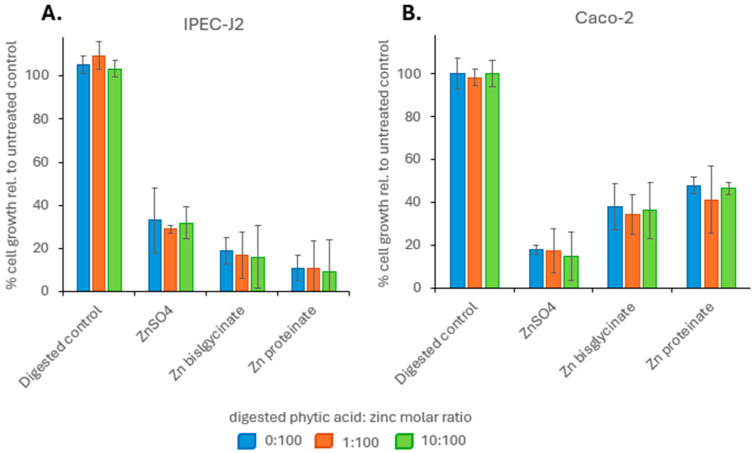
Effect of phytic acid on % cellular growth relative to untreated control (**A**) IPEC-J2 and (**B**) Caco-2 cells (100%) for a 72 h period (*n* = 4). Samples digested with three different phytic acids: zinc molar ratios were used; 0:1 (blue), 1:100 (orange), 1:10 (green). Each bar represents growth relative to untreated control cells.

To determine if phytic acid resulted in a difference in the bioavailability of the zinc compounds, the toxicity and cellular uptake were investigated using two intestinal epithelial cell lines: IPEC-J2 and Caco-2. To remove the influence of bioaccessibility, zinc digests were evaluated at 100 μM zinc. Looking at toxicity, when IPEC-J2 cells were exposed for 72 h to 100 μM of digested ZnSO_4_, Zn bisglycinate or Zn proteinate, there was a decrease in cell growth of 67%, 81%, and 89%, respectively, relative to untreated cells. Cellular toxicity of zinc sources digested with phytic acid showed no change at 100 µM when compared to the toxicity of digested zinc without phytic acid (Figure 6A). In a similar manner, when Caco-2 cells were exposed for 72 h to 100 μM of digested ZnSO_4_, Zn bisglycinate or Zn proteinate, there was a decrease in cell growth of 82%, 62%, and 52%, respectively, relative to untreated cells. Again, there was no change in cellular toxicity of zinc digested with phytic acid compared to those digested without phytic acid (Figure 6B).

**Figure 7:**
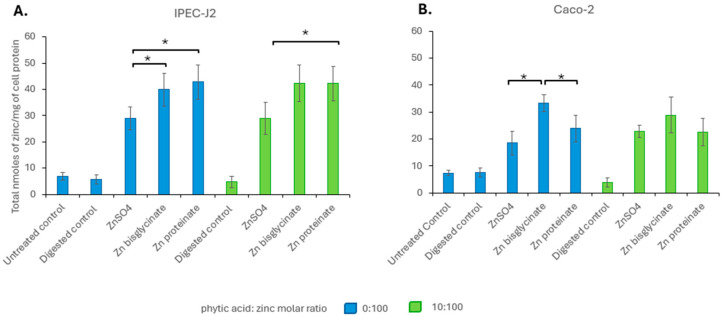
Intracellular zinc levels in nanomoles per milligram of cell protein in (**A**) IPEC-J2 and (**B**) Caco-2 after a 2 h exposure to 100 μM of zinc following *in vitro* digestion in the absence (blue) or presence (green) of phytic acid (*n* = 3). * represents a significant difference between the two data points indicated where *p* ≤ 0.05 and is detailed in Supplementary Table S13.

To assess bioavailability, we investigated the uptake in the cell lines IPEC-J2 and Caco-2. There was no significant difference in uptake for each of the different zinc sources, on an equimolar basis, in the presence or absence of phytate in IPEC-J2 cells (Figure 7A). Similarly, in Caco-2, there were no significant differences in uptake between digests of the same zinc source in the presence or absence of phytic acid (Figure 7B). This suggested that the inhibitory effect of phytic acid on zinc absorption was limited to bioaccessibility, rather than bioavailability. It is also clear that there were some differences in uptake depending on the zinc source and the cell line.

**Figure 8:**
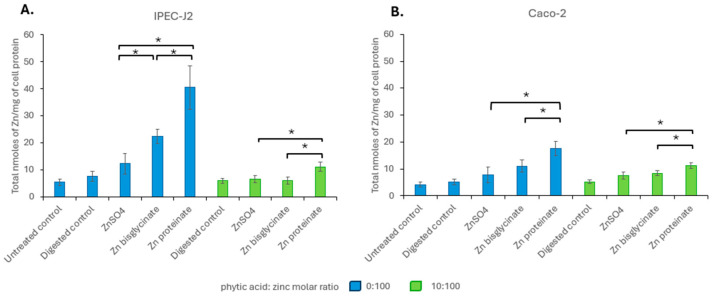
Intracellular zinc levels in nanomoles per milligram of cell protein in (**A**) IPEC-J2 and (**B**) Caco-2 after a 2 h exposure to zinc digests at concentrations reflective of their recovery following *in vitro* digestion in the absence (blue) or presence (green) of phytic acid (*n* = 3). * represents a significant difference between the two data points indicated where *p* ≤ 0.05 and is detailed in Supplementary Table S16.

When comparing zinc uptake after exposure to concentrations reflective to their recovery after digestion, without phytic acid, Zn proteinate showed significantly higher uptake than ZnSO_4_ (*p* < 0.02 for IPEC-J2 and Caco-2) and Zn bisglycinate (*p* < 0.05 for IPEC-J2 and Caco-2) (Figure 8). Uptake of Zn bisglycinate was only significantly higher than ZnSO_4_ for IPEC-J2 and only without phytic acid (*p* < 0.01). Even after digestion with phytic acid, the two cell lines continued to show significantly greater uptake (bioavailability) of Zn proteinate compared to ZnSO_4_ (*p* < 0.03 for both cell lines) (Figure 8).

## 4. Discussion

Zinc is the second-most important trace metal present in the human body with roughly 10% of the proteome having a requirement for or an ability to bind zinc. It plays a role in cell signalling, growth, and differentiation, as well as the functioning of the immune system. It is estimated that 20% of people may be deficient in zinc, including those from third-world countries that are heavily reliant on plant-based diets, as well as vegetarians, vegans, athletes, and the elderly. Deficiency in zinc absorption in the small intestine is related not only to the level of zinc digested but other nutrients that may act as antagonists like phytic acid. It has long been recognised that chelated zinc supplements perform better than inorganic zinc forms (ZnO and ZnSO_4_) in improving crop and animal health and reducing environmental pollution [37]. In both humans and pigs, zinc supplementation reduced inflammatory response, infections, and gastrointestinal disturbances [5,38].

A variety of methods are currently employed to increase the bioaccessibility of nutrients, including zinc, such as the biofortification of grains, vegetable, and fruit crops, foliar spraying, and organic soil fertilisers [39,40,41,42,43]. An alternative approach has been the use of organic sources or zinc rather than inorganic sources. Bioavailability studies *in vivo* with chelated and inorganic sources in humans have also revealed that supplementation of chelated forms (zinc citrate and zinc gluconate) resulted in higher zinc uptake than supplementation with inorganic zinc sources (zinc oxide) [44]. In an *in vitro* digestion study, zinc diglycinate was found to have the highest bioaccessibility (above zinc gluconate, zinc picolinate, and zinc citrate sources), while ZnSO_4_ had the lowest [45] and ZnYeast was more bioavailable following uptake by human enterocytes than ZnSO_4_ [46]. It was also observed that *in vivo* supplementation with a zinc methionine chelate was more effective in contributing to daily weight gain of lambs [46] and increased shell thickness of broiler chicken eggs [47] when compared to inorganic zinc, ZnSO_4_ [47,48]. However, it was noted in RTgutGC cells *in vitro* that despite intracellular accumulation in identical amounts, Zn Bioplex had a lower bioreactivity than ZnSO_4_ and resulted in a slower intracellular release of labile zinc and a reduced intracellular metal detoxification response [49].

In the current study, following *in vitro* digestion, Zn bisglycinate (28%) had higher bioaccessibility than ZnSO_4_ (24%) (Figure 1). Furthermore, Zn proteinate (42%) had the highest bioaccessibility of the three zinc compounds tested and was significantly higher than ZnSO_4_ (*p* < 0.01) or Zn bisglycinate (*p* < 0.01). This demonstrates that chelated forms of zinc were more bioaccessible than inorganic zinc *in vitro*, reflecting *in vivo* findings. Moreover, there were differences in bioaccessibility between the zinc chelates, Zn bisglycinate, and Zn proteinate. These results correlate with the above-mentioned *in vivo* and *in vitro* studies where organic and chelated zinc sources were more bioaccessible or bioavailable.

Another method to increase the bioaccessibility of zinc is to minimise the effect of dietary antagonists such as phytic acid, iron, copper, and calcium [7,8,9]. Phytic acid, the primary phosphorus store in plant foods like grains, legumes, and nuts, reduces the bioavailability of divalent metals like zinc, manganese, magnesium, and calcium *in vivo* by forming stable complexes at acidic pHs, especially during gastric digestion [10]. Monogastric animals such as pigs and humans lack the gastrointestinal enzymes required to break down phytic acid [14]. Thus, there can be a significant decrease in bioavailability of dietary phosphorus and micronutrients, which is a particular problem for those on phytic acid-rich diets [15,18,19]. In addition, it presents an agricultural challenge as unabsorbed minerals are excreted as environmental pollutants [20]. Traditional techniques such as soaking, milling, dehulling, and heating can effectively reduce phytic acid levels [50,51], but biological methods such as fermentation and phytase hydrolysis present an advantage over traditional methods, having higher specificity for phytic acid without compromising the nutritional integrity of the diet [52].

The relationship between the bioaccessibility and bioavailability of three different zinc sources in the presence of the antagonist phytic acid was investigated using an *in vitro* system. The aim was to determine at what stage or stages the loss in zinc recovery occurred. Following completion of the standardised static *in vitro* digestion of inorganic and chelated zinc, the presence of phytic acid reduced the recovery of all three zinc sources at the molar ratio of 10:100 phytic acid/zinc (Figure 1). The recovery of inorganic zinc was significantly affected at the 2:100 molar ratio (*p* < 0.01), with an increasing loss at higher phytic acid/zinc molar ratios. The bioaccessibility of chelated zinc sources were not impacted until molar ratios 4:100 (*p* < 0.05) and 10:100 (*p* < 0.01) (Figure 1). Zn proteinate had significantly higher bioaccessibility at all phytic acid/zinc molar ratios tested compared to both ZnSO_4_ and Zn bisglycinate (*p* < 0.01) (Supplementary Tables S1 and S4).

To confirm the role of phytic acid in reducing zinc bioaccessibility, phytase was introduced to the digestion process to reverse this reduction. The inclusion of phytase in animal feeds has been observed to increase zinc digestibility and retention in broiler chickens [53] and swine [54,55]. Furthermore, phytase has also been noted to increase zinc solubility *in vitro* [56,57]. There are two main sources of phytase: microbial and plant. Both *Aspergillus niger* phytase and wheat phytase were shown to degrade phytic acid in cereals under simulated gastric conditions [58]. Moreover, microbial phytases are reported to be more efficient than plant phytases, resulting in a larger dephosphorylation at a gastric pH of three, releasing zinc and iron from the cereals [58,59].

The phytase from *Aspergillus niger* when included in the digestion process restored the zinc levels to that of each zinc digested without phytic acid (Figure 4). The dephosphorylation of phytic acid by the phytase was observed by the absence of phytic acid bands in samples prior to and after digestion (Figure 2 and Figure 3). It has also observed *in vivo* that inclusion of *Aspergillus niger* phytase results in an increase in the digestibility and bioavailability of zinc [60,61]. Although wheat phytase was shown to also break down phytic acid (Figure 2), it failed to ameliorate the effect of phytic acid on the recovery of zinc after digestion (Figure 5). Zn bisglycinate was less affected by the wheat phytase but the incorporation of phytic acid and wheat phytase resulted in similar yields to the ZnSO_4_ of 14% and 12% of starting zinc, respectively, (Figure 5). The bioaccessibility of Zn proteinate was also affected by the wheat phytase itself; however, it was still significantly higher than ZnSO_4_ (*p* < 0.01) and Zn bisglycinate (*p* < 0.05). Moreover, inclusion of wheat phytase did improve the recovery of Zn proteinate when digested in conjunction with phytic acid, with substantial differences observed compared to ZnSO_4_ (*p* < 0.05) and Zn bisglycinate (*p* < 0.01) (Figure 5).

Why did wheat phytase not restore the recovery of zinc in the way that *Aspergillus niger* phytase did, and why did it have as negative an impact as phytic acid itself on zinc recovery? The reason may be due to the structurally distinct classes of phytases that cleave phosphate groups from phytic acid and in the way that cleavage is achieved. Both wheat phytase and *Aspergillus niger* phytase belong to the histidine acid phosphatases (HAPs). Here, the histidine in the catalytic site causes a nucleophilic attack on a phosphate group of phytic acid. This is followed by hydrolysis of the phosphate–histidine bond [62]. In the case of *Aspergillus niger* phytase, six amino acid residues (K91, K94, E228, D262, K300 and K301) in the substrate specificity site of PhyA (the phytase used in this study) encircle the active site [63], making it very specific for negatively charged phytic acid [64]. This is noted in the very low K_m_ of 0.9 µmol/L that *Aspergillus niger* phytase has for phytic acid [65]. The K_m_ of wheat phytase for phytic acid is much higher at 838 µmol/L [66].

There are also tantalising suggestions from the literature on the interaction of phytases and zinc. When examining the use of phytase to release phosphates from phytic acid, it has been suggested that zinc inhibits phytase activity *in vitro* by interacting with the histidine residue in the phytase catalytic site [67]. Zinc was also seen to inhibit the activity of *Escherichia coli* phytase by 80% [68,69], while *Aspergillus niger* phytase was only reduced by 30% [70]. Given that the imidazole ring of histidine binds zinc ions strongly, it is possible that zinc can sterically hinder phytic acid access to the catalytic site of phytase. The substrate specificity site of *Aspergillus niger* phytase may reduce the ability of zinc to interact with histidine in the catalytic site by providing an optimised binding for negatively charged phytic acid.

As bioaccessibility and bioavailability can affect absorption, the impact of phytic acid on the bioavailability is measured by cellular uptake of the three zinc sources. Both toxicity and uptake showed no change at equimolar concentrations of zinc digested with or without phytic acid for each of the zinc sources (Figure 6; toxicity, Figure 7; uptake), indicating that phytic acid was not acting at the level of uptake. It has been noted that zinc proteinates are not only more bioaccessible than their inorganic and chelated zinc counterparts, but they also have higher bioavailability in broiler chicks [71,72,73,74] and in calves [75]. For both IPEC-J2 and Caco-2 cells, while there were differences in the cellular uptake of the three zinc sources at equimolar concentrations, it was the uptake at concentrations reflecting bioaccessibility that were more representative of the *in vivo* absorption. Here, the uptake of Zn proteinate was highest both with and without phytic acid, showing its increased bioavailability even following digestion with phytic acid (Figure 8).

## 5. Conclusions

The use of an *in vitro* study allowed both bioaccessibility and bioavailability to be successfully distinguished through a standardised *in vitro* digestion (bioaccessibility) and cell-based zinc uptake (bioavailability). Bioavailability was not affected at equimolar zinc concentrations. Rather, phytic acid affects zinc’s bioaccessibility during digestion, with a knock-on effect on bioavailability depending on the zinc source. In particular, Zn proteinate was observed to have significantly higher bioaccessibility and bioavailability of the zinc sources investigated after digestion, both with and without phytic acid.

## Data Availability

The original contributions presented in this study are included in the article/Supplementary Materials. Further inquiries can be directed to the corresponding author.

## Supplementary Materials

The following supporting information can be downloaded at https://www.mdpi.com/article/10.3390/nu18010046/s1. Figure S1: Validation of colourimetric zinc assay values through flame atomic absorption spectrometry. Table S1: Statistic appraisal of impact of phytic acid on zinc bioaccessibility: Student’s *t*-test with unequal variance and a two-tailed distribution (*n* = 5). Table S2: Statistic appraisal of impact of phytic acid on zinc bioaccessibility using ANOVA: Two Factor with Replication (*n* = 5). Table S3: Statistic appraisal of impact of phytic acid on zinc bioaccessibility using Tukey’s multiple comparisons test within zinc sources following ANOVA: Two Factor with Replication (*n* = 5). Table S4: Statistic appraisal of impact of phytic acid on zinc bioaccessibility using Tukey’s multiple comparisons test between zinc sources following ANOVA: Two Factor with Replication (*n* = 5). Figure S2: PAGE analysis showing effect of treatment of Phytic acid standards with A. phytase from *Aspergillus niger* at 37 °C, B. phytase from *Aspergillus niger* at 55 °C, C. phytase from wheat at 37 °C, D. with phytase from wheat at 55 °C. (*n* = 3). Figure S3: PAGE analysis of zinc sources digested in vitro with (+) or without phytic acid (−) in a molar ratio of 10:100 phytic acid: zinc. Representative blot shown (*n* = 3). Table S5: Statistic appraisal of impact of phytase (*Aspergillus niger*) activity on zinc bioaccessibility: Student’s *t*-test with unequal variance and a two-tailed distribution (*n* = 3). Table S6: Statistic appraisal of impact of phytase (*Aspergillus niger*) activity on zinc bioaccessibility: ANOVA: Two factor with replicates (*n* = 3). Table S7: Statistic appraisal of impact of phytase (*Aspergillus niger*) activity on zinc bioaccessibility Tukey’s multiple comparisons test within zinc sources following ANOVA: Two Factor with Replication (*n* = 3). Table S8: Statistic appraisal of impact of phytase (*Aspergillus niger*) activity on zinc bioaccessibility Tukey’s multiple comparisons test between zinc sources following ANOVA: Two Factor with Replication (*n* = 3). Table S9: Statistic appraisal of impact of phytase (wheat) activity on zinc bioaccessibility: Student’s *t*-test with unequal variance and a two-tailed distribution Table S10: Statistic appraisal of impact of phytase (wheat) activity on zinc bioaccessibility: ANOVA: Two factor with replicates (*n* = 3). Table S11: Statistic appraisal of impact of phytase (wheat) activity on zinc bioaccessibility Tukey’s multiple comparisons test within zinc sources following ANOVA: Two Factor with Replication (*n* = 3). Table S12: Statistic appraisal of impact of phytase (wheat) activity on zinc bioaccessibility Tukey’s multiple comparisons test between zinc sources following ANOVA: Two Factor with Replication (*n* = 3). Table S13: Student’s *t*-test with unequal variance and a two-tailed distribution (*n* = 3). Statistic appraisal of zinc uptake of different zinc sources at 100 μM: in A. IPEC-J2 and B. Caco-2 cells Table S14: ANOVA: Two factor with replicates (*n* = 3). Statistic appraisal of zinc uptake of different zinc sources at 100 μM in A. IPEC-J2 and B. Caco-2 cells Table S15: ANOVA: Two factor with replicates (*n* = 3). Statistical appraisal of zinc uptake of different zinc sources at 100 μM in A. IPEC-J2 and B. Caco-2 cells. Table S16: Statistic appraisal of zinc uptake of different zinc sources: *p*-values of statistical difference in uptake of zinc at concentrations reflective of recovery following *in vitro* digestion in A. IPEC-J2 and B. Caco-2 cells. Table S17: ANOVA: Two factor with replicates (*n* = 3). Statistic appraisal of zinc uptake of different zinc sources at concentrations reflective of recovery following *in vitro* digestion in A. IPEC-J2 and B. Caco-2 cells. Table S18: Tukey’s test following ANOVA: Two factor with replicates (*n* = 3). Statistic appraisal of zinc uptake of different zinc sources at concentrations reflective of recovery following *in vitro* digestion in A. IPEC-J2 and B. Caco-2 cells.
