# Supplementary material for: The Antagonistic Influence of Phytic Acid on Zinc Absorption: An In Vitro Comparison of Inorganic and Chelated Trace Mineral Sources
Source: Nutrients. 2025 Dec 22;18(1):46. doi: 10.3390/nu18010046 (PMC12788162; doi:10.3390/nu18010046)
Supplement: Supplementary file 1 [file nutrients-18-00046-s001.zip › nutrients-3943526-supplementary.pdf]

Supplementary data

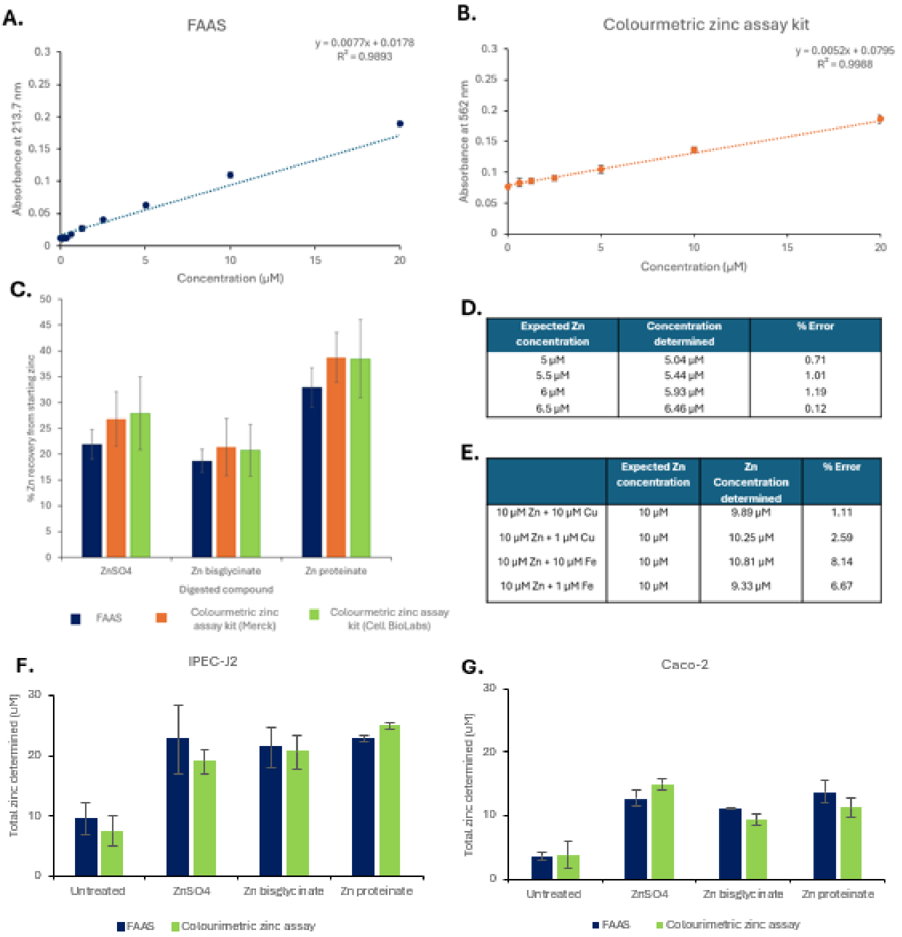

D.

Expected Zn concentration	Concentration determined	% Error
5 µM	5.04 µM	0.71
5.5 µM	5.44 µM	1.01
6 µM	5.93 µM	1.19
6.5 µM	6.46 µM	0.12

E.

	Expected Zn concentration	Zn Concentration determined	% Error
10 µM Zn + 10 µM Cu	10 µM	9.89 µM	1.11
10 µM Zn + 1 µM Cu	10 µM	10.25 µM	2.59
10 µM Zn + 10 µM Fe	10 µM	10.81 µM	8.14
10 µM Zn + 1 µM Fe	10 µM	9.33 µM	6.67

Figure S1 – Validation of colourimetric zinc assay values through flame atomic absorption spectrometry. Zinc standards (0.5 g/0.5 L, Merck®, Darmstadt, Germany) were prepared (0-20 µM) with UHP and analysed using both **A.** flame atomic absorption spectrometry (FAAS) and **B.** colourimetric zinc assay kits. FAAS was completed using an air-acetylene flame with a deuterium background correction (λ = 213.9 nm) on Varian SpectraAA 50 in the School of Chemical Sciences, Dublin City University, Ireland. The lower limit of detection and quantification for the FAAS were 0.617 µM and 1.87 µM, respectively. For the colourimetric zinc assay kit, the lower limit of detection and quantification were 0.78 µM and 2.37 µM, respectively. **C.** A comparison of the quantification of digested zinc compounds using FAAS and two colourimetric assay kits, revealed no statistically significant differences in the obtained concentrations ($p > 0.05$). **D.** A comparison of the quantification of zinc using the colourimetric assay kit against the concentration expected, indicating the sensitivity of colourimetric kit **E.** There was minimal cross reactivity observed for other divalent metals such as copper or iron using either method with a percentage error less than 9 % observed . For example, concentrations of 1 µM and 10 µM for both iron and copper did not result in a detectable colourimetric signal and agreed when combined with zinc, the zinc concentration determined agreed with expected value within 9 % (n =4)**.** A comparison of the quantification of zinc concentrations in **F.** IPEC-J2 cell lysates and **G.** Caco-2 cell lysates using FAAS and two colourimetric assay kits, revealed no statistically significant differences in the obtained concentrations ($p > 0.05$).

Table S1 – Statistic appraisal of impact of phytic acid on zinc bioaccessibility: Student's t-test with unequal variance and a two-tailed distribution (n=5). **A.** statistical difference compared to the 0:100 phytic acid: zinc molar ratio for each zinc. For each zinc, the recovery (average +/- standard deviation) is shown without phytic acid compared to a specific molar ratio of phytic acid: zinc. **B.** Statistical difference between zinc sources at each phytic acid: zinc molar ratio (If in grey, not statistically significant; If in **Bold** p<0.05 and if in ***bold italics*** p < 0.01).

A.

phytic acid: zinc molar ratio	$ZnSO_4$		Zn bisglycinate		Zn proteinate	
	% recovery	p value	% recovery	p value	% recovery	p value
0:1	24 ± 1	0.484	28 ± 3	0.92	42 ± 1	0.49
1:100	22 ± 4		28 ± 4		41 ± 1	
0:1	24 ± 1	**6 x 10^{-3}**	28 ± 3	0.12	42 ± 1	0.12
2:100	16 ± 2		26 ± 1		40 ± 7	
0:1	24 ± 1	**1.5 x 10^{-2}**	28 ± 3	**3 x 10^{-3}**	42 ± 1	**4 x 10^{-3}**
4:100	18 ± 2		21± 1		31 ± 3	
0:1	24 ± 1	**1.2 x 10^{-5}**	28 ± 3	**6 x 10^{-3}**	42 ± 1	**4.5 x 10^{-3}**
10:100	9 ± 1		9 ± 5		17 ± 1	

B.

phytic acid: zinc molar ratio	$ZnSO_4$ / Zn bisglycinate		$ZnSO_4$ / Zn proteinate		Zn bisglycinate / Zn proteinate	
	% recovery	p value	% recovery	p value	% recovery	p value
0:1	24 ± 1	0.12	24 ± 1	***2.2 x 10^{-7}***	28 ± 3	***2.2 x 10^{-4}***
	28 ± 3		42 ± 1		42 ± 1	
1:100	22 ± 4	**5.6 x 10^{-2}**	28 ± 3	***4.8 x 10^{-3}***	28 ± 4	***9.8 x 10^{-3}***
	28 ± 4		41 ± 1		41 ± 1	
2:100	16± 2	**6.1 x 10^{-3}**	28 ± 3	***5 x 10^{-3}***	26 ± 1	***3.2 x 10^{-2}***
	26 ± 1		42 ± 1		42 ± 1	
4:100	18 ± 2	**2.4 x 10^{-2}**	28 ± 3	***3.2 x 10^{-4}***	21 ± 1	***5.3 x 10^{-3}***
	21 ± 1		31± 3		31 ± 3	
10:100	9 ± 1	0.28	9 ± 1	***2.5 x 10^{-4}***	9 ± 5	***2.3 x 10^{-3}***
	9 ± 5		17 ± 1		17 ± 1	

Table S2 – Statistic appraisal of impact of phytic acid on zinc bioaccessibility using ANOVA: Two Factor with Replication (n=5). (If in grey, not statistically significant; if in **Bold** $p < 0.05$ and if in ***bold italics,*** $p < 0.01$).

Anova: Two-Factor With Replication				
SUMMARY	$ZnSO_4$	Zn bisglycinate	Zn proteinate	Total
0:100				
Count	5	5	5	15
Sum	124.88	141.06	211.61	477.55
Average	24.98	28.21	42.32	31.84
Variance	6.94	10.35	4.19	66.91
1:100				
Count	5	5	5	15
Sum	112.17	144.09	203.69	459.95
Average	22.43	28.82	40.74	30.66
Variance	25.11	7.13	2.14	71.47
2:100				
Count	5	5	5	15
Sum	83.08	127.10	185.34	395.52
Average	16.62	25.42	37.07	26.37
Variance	8.31	0.52	23.70	84.47
4:100				
Count	5	5	5	15
Sum	82.98	108.26	154.99	346.23
Average	16.60	21.65	31.00	23.08
Variance	7.21	8.08	19.74	48.15
10:100				
Count	5	5	5	15
Sum	41.33	46.15	82.45	169.93
Average	8.27	9.23	16.49	11.33
Variance	1.28	7.65	4.55	18.29
Total				
Count	25	25	25	
Sum	444.43	566.66	838.08	
Average	17.78	22.67	33.52	
Variance	42.88	59.32	100.42	

ANOVA						
Source of Variation	*SS*	*df*	*MS*	*F*	*P-value*	*F crit*
Sample	4060.32	4	1015.08	111.21	***4.9E-27***	2.53
Columns	3247.61	2	1623.81	177.91	***6E-26***	3.15
Interaction	254.87	8	31.86	3.49	***0.0023***	2.10
Within	547.63	60	9.13			
Total	8110.43	74				

Table S3 – Statistic appraisal of impact of phytic acid on zinc bioaccessibility using Tukey's multiple comparisons test within zinc sources following ANOVA: Two Factor with Replication (n=5). (If in grey, not statistically significant; if in **Bold** $p < 0.05$ and if in ***bold italics,*** $p < 0.01$).

Tukey's multiple comparisons test	Mean diff.	95.00% CI of diff.	Below threshold?	Summary	Adjusted P Value
**ZnSO$_4$**					
0:100 vs. 1:100	3.97	-1.821 to 9.756	No	ns	0.2139
0:100 vs. 2:100	***10.46***	***4.983 to 15.94***	***Yes***	**	***0.0019***
0:100 vs. 4:100	***9.56***	***3.460 to 15.65***	***Yes***	**	***0.0042***
0:100 vs. 10:100	***17.89***	***12.42 to 23.36***	***Yes***	***	***0.0002***
1:100 vs. 2:100	***6.49***	***1.942 to 11.04***	***Yes***	**	***0.0081***
1:100 vs. 4:100	***5.59***	***0.069 to 11.11***	***Yes***	*	***0.0472***
1:100 vs. 10:100	***13.92***	***9.52 to 18.31***	***Yes***	***	***0.0002***
2:100 vs. 4:100	-0.90	-6.04 to 4.230	No	ns	0.9637
2:100 vs. 10:100	***7.43***	***4.17 to 10.68***	***Yes***	***	***0.0005***
4:100 vs. 10:100	***8.33***	***3.25 to 13.41***	***Yes***	**	***0.0058***
**Zn bisglycinate**					
0:100 vs. 1:100	-0.61	-7.12 to 5.908	No	ns	0.9971
0:100 vs. 2:100	2.79	-3.46 to 9.046	No	ns	0.4319
0:100 vs. 4:100	6.56	-0.01 to 13.22	No	ns	0.0536
0:100 vs. 10:100	***18.98***	***12.39 to 25.57***	***Yes***	****	***<0.0001***
1:100 vs. 2:100	3.40	-1.75 to 8.551	No	ns	0.1861
**1:100 vs. 4:100**	**7.17**	**1.14 to 13.20**	**Yes**	*	**0.021**
1:100 vs. 10:100	***19.59***	***13.65 to 25.53***	***Yes***	****	***<0.0001***
2:100 vs. 4:100	3.77	-1.73 to 9.265	No	ns	0.1662
2:100 vs. 10:100	***16.19***	***10.85 to 21.53***	***Yes***	***	***0.0005***
4:100 vs. 10:100	***12.42***	***6.29 to 18.55***	***Yes***	***	***0.0008***
**Zn proteinate**					
0:100 vs. 1:100	1.59	-2.40 to 5.574	No	ns	0.6407
0:100 vs. 2:100	5.26	-3.96 to 14.47	No	ns	0.2957
**0:100 vs. 4:100**	**11.32**	**2.93 to 19.72**	**Yes**	*	**0.0137**
0:100 vs. 10:100	***25.83***	***21.27 to 30.40***	***Yes***	****	***<0.0001***
1:100 vs. 2:100	3.67	-5.68 to 13.01	No	ns	0.5495
**1:100 vs. 4:100**	**9.74**	**1.25 to 18.23**	**Yes**	*	**0.0297**
1:100 vs. 10:100	***24.25***	***20.12 to 28.37***	***Yes***	****	***<0.0001***
2:100 vs. 4:100	6.07	-4.14 to 16.27	No	ns	0.3219
2:100 vs. 10:100	***20.58***	***11.38 to 29.78***	***Yes***	**	***0.0012***
4:100 vs. 10:100	***14.51***	***6.12 to 22.90***	***Yes***	**	***0.004***

Table S4 – Statistic appraisal of impact of phytic acid on zinc bioaccessibility using Tukey's multiple comparisons test between zinc sources following ANOVA: Two Factor with Replication (n=5). (If in grey, not statistically significant; if in **Bold** $p < 0.05$ and if in ***bold italics,*** $p < 0.01$).

Tukey's multiple comparisons test	Mean diff.	95.00% CI of diff.	Below threshold?	Summary	Adjusted P Value
0:100					
ZnSO4 vs. Zn bisglycinate	-2.06	-8.7 to 4.58	No	ns	0.56
$ZnSO_4$ vs. Zn proteinate	***-16.17***	***-23.33 to -9.01***	***Yes***	**	***0.0029***
Zn bisglycinate vs. Zn proteinate	***-14.11***	***-21.22 to -7.004***	***Yes***	**	***0.0046***
**1:100**					
**$ZnSO_4$ vs. Zn bisglycinate**	**-6.64**	**-12.00 to -1.27**	**Yes**	*	**0.0251**
$ZnSO_4$ vs. Zn proteinate	***-18.55***	***-23.99 to -13.11***	***Yes***	***	***0.0006***
Zn bisglycinate vs. Zn proteinate	***-11.92***	***-15.19 to -8.65***	***Yes***	***	***0.0005***
2:100					
$ZnSO_4$ vs. Zn bisglycinate	***-9.73***	***-13.04 to -6.412***	***Yes***	**	***0.0011***
$ZnSO_4$ vs. Zn proteinate	***-21.38***	***-29.64 to -13.11***	***Yes***	**	***0.0017***
Zn bisglycinate vs. Zn proteinate	***-11.65***	***-18.75 to -4.54***	***Yes***	**	***0.0094***
4:100					
ZnSO4 vs. Zn bisglycinate	-5.06	-11.57 to 1.460	No	ns	0.1045
**$ZnSO_4$ vs. Zn proteinate**	**-14.40**	**-24.36 to -4.448**	**Yes**	*	**0.0147**
**Zn bisglycinate vs. Zn proteinate**	**-9.35**	**-15.97 to -2.722**	**Yes**	*	**0.016**
10:100					
ZnSO4 vs. Zn bisglycinate	-0.97	-7.133 to 5.202	No	ns	0.8483
$ZnSO_4$ vs. Zn proteinate	***-8.23***	***-12.69 to -3.758***	***Yes***	**	***0.0061***
Zn bisglycinate vs. Zn proteinate	***-7.26***	***-11.76 to -2.761***	***Yes***	**	***0.0099***

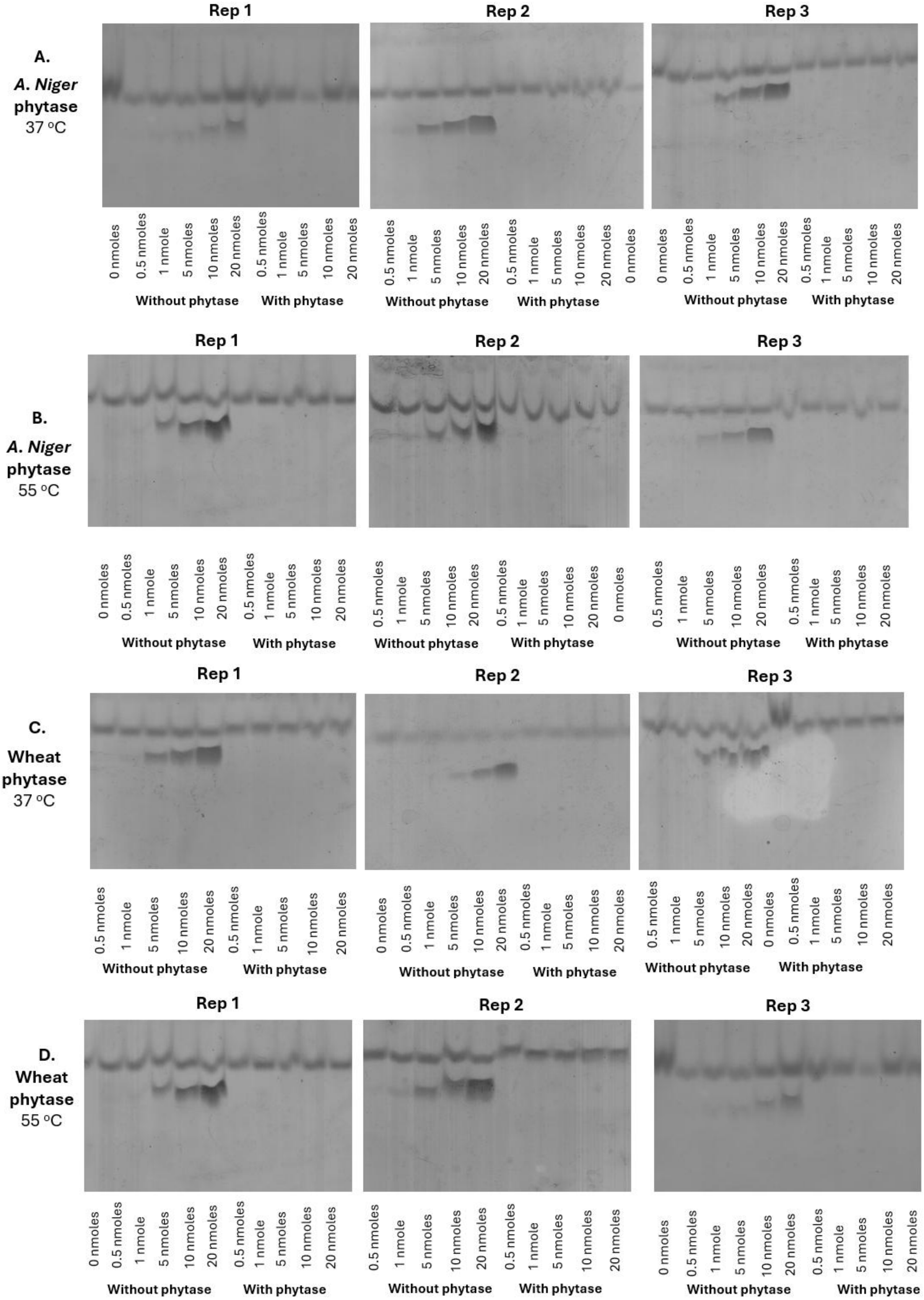

Figure S2 – PAGE analysis showing effect of treatment of Phytic acid standards with **A.** phytase from *Aspergillus niger* at 37 °C, **B.** phytase from *Aspergillus niger* at 55 °C, **C.** phytase from wheat at 37 °C, **D.** with phytase from wheat at 55 °C. (n=3).

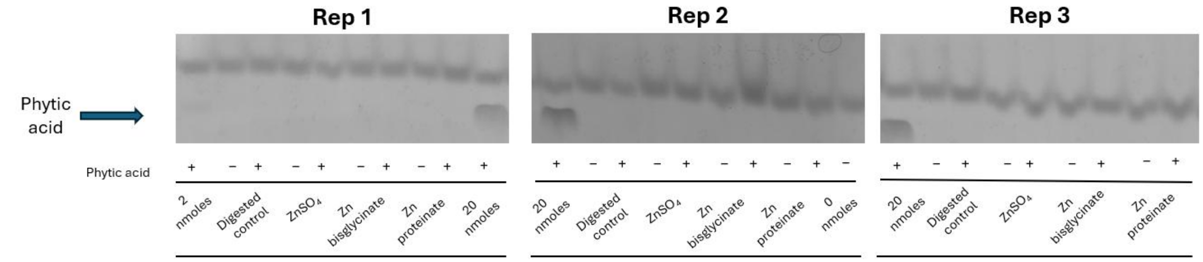

Figure S3 – PAGE analysis of zinc sources digested in vitro with (+) or without phytic acid (−) in a molar ratio of 10:100 phytic acid: zinc. Representative blot shown (n=3).

Table S5 - Statistic appraisal of impact of phytase (*Aspergillus niger*) activity on zinc bioaccessibility: Student's t-test with unequal variance and a two-tailed distribution (n=3). **A.** compared to the 0:100 phytic acid: zinc molar ratio for each zinc. For each zinc, the recovery (average +/- standard deviation) is shown for each zinc without phytic acid compared to a specific molar ratio of phytic acid: zinc. **B.** Statistical difference between zinc sources under each condition. (If in grey, not statistically significant; If in Bold $p \leq 0.05$ and if in bold italics, $p < 0.01$).

A.

	$ZnSO_4$		Zn bisglycinate		**Zn proteinate**	
Condition	% recovery	p value	% recovery	p value	% recovery	p value
**(i)**	27 ± 1	***8.1×10^{-4}***	28 ± 3	**0.02**	36 ± 4	**0.01**
**(ii)**	12 ± 1		9 ± 2		19 ± 1	

B.

	$ZnSO_4$		Zn bisglycinate	
	Zn proteinate		Zn proteinate	
Condition	% recovery	p value	% recovery	p value
**(i)**	27 ± 1	**0.05**	28 ± 3	***6.7×10^{-3}***
	36 ± 4		36 ± 4	
**(ii)**	12 ± 1	***2×10^{-3}***	9 ± 2	***1.4×10^{-3}***
	19 ± 1		19 ± 1	
**(iii)**	24 ± 6	**0.05**	17 ± 1	***4.9×10^{-5}***
	38 ± 1		38 ± 1	
**(iv)**	24 ± 4	**0.04**	20 ±1	**0.02**
	34 ± 5		34 ± 5	

Table S6 – Statistic appraisal of impact of phytase (*Aspergillus niger*) activity on zinc bioaccessibility: ANOVA: Two factor with replicates (n=3). (If in grey, not statistically significant; if in **Bold** $p < 0.05$ and if in ***bold italics,*** $p < 0.01$).

Anova: Two-Factor With Replication				
SUMMARY	$ZnSO_4$	Zn bisglycinate	Zn proteinate	Total
(i)				
Count	3	3	3	9
Sum	81.22	60.44	109.01	250.68
Average	27.07	20.15	36.34	27.85
Variance	0.40	13.56	15.73	56.92
(ii)				
Count	3	3	3	9
Sum	35.40	26.08	56.79	118.27
Average	11.80	8.69	18.93	13.14
Variance	1.64	2.48	1.06	21.96
(iii)				
Count	3	3	3	9
Sum	73.24	50.49	113.14	236.88
Average	24.41	16.83	37.71	26.32
Variance	34.07	1.93	1.24	93.13
(iv)				
Count	3	3	3	9
Sum	71.00	58.25	100.65	229.90
Average	23.67	19.42	33.55	25.54
Variance	12.34	0.38	26.83	49.31
Total				
Count	12	12	12	
Sum	260.86	195.26	379.60	
Average	21.74	16.27	31.63	
Variance	46.47	25.88	69.29	

ANOVA						
Source of Variation	*SS*	*df*	*MS*	*F*	*P-value*	*F crit*
**Sample**	**1242.52**	**3**	**414.17**	**44.51**	***5.8E-10***	**3.01**
**Columns**	**1454.98**	**2**	**727.49**	**78.18**	***3.1E-11***	**3.40**
Interaction	92.26	6	15.38	1.65	0.176252	2.51
Within	223.33	24	9.31			
Total	3013.08	35				

Table S7 – Statistic appraisal of impact of phytase (*Aspergillus niger*) activity on zinc bioaccessibility Tukey's multiple comparisons test within zinc sources following ANOVA: Two Factor with Replication (n=3). (If in grey, not statistically significant; if in **Bold** p < 0.05 and if in ***bold italics,*** p < 0.01).

Tukey's multiple comparisons test	Mean diff.	95.00% CI of diff.	Below threshold?	Summary	Adjusted P Value
$ZnSO_4$					
(i) vs. (ii)	***15***	***8.4 to 22***	***Yes***	****	***<0.0001***
(i) vs. (iii)	2.7	-4.2 to 9.5	No	ns	0.7117
(i) vs. (iv)	3.4	-3.5 to 10	No	ns	0.531
(ii) vs. (iii)	***-13***	***-19 to -5.7***	***Yes***	***	***0.0002***
(ii) vs. (iv)	***-12***	***-19 to -5.0***	***Yes***	***	***0.0004***
(iii) vs. (iv)	0.75	-6.1 to 7.6	No	ns	0.9904
Zn bisglycinate					
(i) vs. (ii)	***11***	***4.6 to 18***	***Yes***	***	***0.0006***
(i) vs. (iii)	3.3	-3.6 to 10	No	ns	0.5527
(i) vs. (iv)	0.73	-6.1 to 7.6	No	ns	0.991
**(ii) vs. (iii)**	**-8.1**	**-15 to -1.3**	**Yes**	*	**0.0161**
(ii) vs. (iv)	***-11***	***-18 to -3.9***	***Yes***	**	***0.0013***
(iii) vs. (iv)	-2.6	-9.5 to 4.3	No	ns	0.729
Zn proteinate					
(i) vs. (ii)	***17***	***11 to 24***	***Yes***	****	***<0.0001***
(i) vs. (iii)	-1.4	-8.2 to 5.5	No	ns	0.9449
(i) vs. (iv)	2.8	-4.1 to 9.7	No	ns	0.6811
(ii) vs. (iii)	***-19***	***-26 to -12***	***Yes***	****	***<0.0001***
(ii) vs. (iv)	***-15***	***-21 to -7.7***	***Yes***	****	***<0.0001***
(iii) vs. (iv)	4.2	-2.7 to 11	No	ns	0.3594

Table S8 – Statistic appraisal of impact of phytase (*Aspergillus niger*) activity on zinc bioaccessibility Tukey's multiple comparisons test between zinc sources following ANOVA: Two Factor with Replication (n=3). (If in grey, not statistically significant; if in **Bold** p<0.05 and if in ***bold italics,*** p<0.01).

Tukey's multiple comparisons test	Mean diff.	95.00% CI of diff.	Below threshold?	Summary	Adjusted P Value
		(i)			
**$ZnSO_4$ vs. Zn bisglycinate**	**6.9**	**0.71 to 13**	**Yes**	*****	**0.027**
$ZnSO_4$ vs. Zn proteinate	***-9.3***	***-15 to -3.0***	***Yes***	********	***0.003***
Zn bisglycinate vs. Zn proteinate	***-16***	***-22 to -10***	***Yes***	**********	***<0.0001***
		(ii)			
$ZnSO_4$ vs. Zn bisglycinate	3.1	-3.1 to 9.3	No	ns	0.4376
**$ZnSO_4$ vs. Zn proteinate**	**-7.1**	**-13 to -0.91**	**Yes**	*****	**0.0225**
Zn bisglycinate vs. Zn proteinate	***-10***	***-16 to -4.0***	***Yes***	********	***0.0011***
		(iii)			
**$ZnSO_4$ vs. Zn bisglycinate**	**7.6**	**1.4 to 14**	**Yes**	*****	**0.0149**
$ZnSO_4$ vs. Zn proteinate	***-13***	***-20 to -7.1***	***Yes***	**********	***<0.0001***
Zn bisglycinate vs. Zn proteinate	***-21***	***-27 to -15***	***Yes***	**********	***<0.0001***
		(iv)			
$ZnSO_4$ vs. Zn bisglycinate	4.3	-2.0 to 10	No	ns	0.2234
$ZnSO_4$ vs. Zn proteinate	***-9.9***	***-16 to -3.7***	***Yes***	********	***0.0016***
Zn bisglycinate vs. Zn proteinate	***-14***	***-20 to -7.9***	***Yes***	**********	***<0.0001***

Table S9– Statistic appraisal of impact of phytase (wheat) activity on zinc bioaccessibility: Student's t-test with unequal variance and a two-tailed distribution **A.** compared to the 0:100 phytic acid: zinc molar ratio for each zinc **B**. Statistical difference between zinc sources under each condition. (If in **Bold** $p \leq 0.05$ and if in ***bold italics***, $p < 0.01$) (n=3).

A.

phytic acid: zinc molar ratio	$ZnSO_4$		Zn bisglycinate		Zn proteinate	
	% recovery	p value	% recovery	p value	% recovery	p value
**(i)**	25 ± 3	***2.8 x 10^{-4}***	28 ± 3	***3.9 x 10^{-3}***	36 ± 4	***2.7 x 10^{-4}***
**(ii)**	10 ± 1		11 ± 1		18 ± 1	
**(i)**	25 ± 3	***6.6 x 10^{-5}***	28 ± 3	***5.6 x 10^{-3}***	36 ± 4	**0.02**
**(iii)**	10 ± 2		17 ± 2		29 ± 5	
**(i)**	25 ± 3		28 ± 3	***6.1 x 10^{-3}***	36 ± 4	***8.2 x 10^{-4}***
**(iv)**	13 ± 4	***9.1 x 10^{-3}***	11 ± 1		23 ± 1	

B.

phytic acid: zinc molar ratio	$ZnSO_4$ / Zn proteinate		Zn bisglycinate / Zn proteinate	
	% recovery	p value	% recovery	p value
**(i)**	27 ± 1	***9.6 x 10^{-3}***	28 ± 3	***1 x 10^{-2}***
	36 ± 4		36 ± 4	
**(ii)**	12 ± 1	***3.9 x 10^{-4}***	9 ± 2	***7.2 x 10^{-4}***
	19 ± 1		19 ± 1	
**(iii)**	24 ± 6	***7.6 x 10^{-3}***	17 ± 1	***8.5 x 10^{-3}***
	38 ± 1		38 ± 1	
**(iv)**	24 ± 4	**0.03**	20 ±1	**1.7 x 10^{-3}**
	34 ± 5		34 ± 5	

Table S10 – Statistic appraisal of impact of phytase (wheat) activity on zinc bioaccessibility: ANOVA: Two factor with replicates (n=3). (If in grey, not statistically significant; if in **Bold** $p < 0.05$ and if in ***bold italics,*** $p < 0.01$).

Anova: Two-Factor With Replication				
SUMMARY	$ZnSO_4$	Zn bisglycinate	Zn proteinate	Total
(i)				
Count	3	3	3	9
Sum	70.19	85.33	115.20	270.71
Average	23.40	28.44	38.40	30.08
Variance	7.15	7.29	15.40	51.17
(ii)				
Count	3	3	3	9
Sum	30.02	32.32	53.46	115.81
Average	10.01	10.77	17.82	12.87
Variance	0.56	0.87	0.82	14.47
(iii)				
Count	3	3	3	9
Sum	27.36	49.65	80.59	157.60
Average	9.12	16.55	26.86	17.51
Variance	2.48	2.54	14.71	64.48
(iv)				
Count	3	3	3	9
Sum	39.52	34.16	69.97	143.66
Average	13.17	11.39	23.32	15.96
Variance	12.72	0.22	1.74	34.75
Total				
Count	12	12	12	
Sum	167.09	201.47	319.21	
Average	13.92	16.79	26.60	
Variance	39.27	56.86	67.88	

ANOVA						
Source of Variation	*SS*	*df*	*MS*	*F*	*P-value*	*F crit*
**Sample**	**1545.81**	**3**	**515.27**	**92.97**	***2.4E-13***	**3.01**
**Columns**	**1060.74**	**2**	**530.37**	**95.70**	***3.7E-12***	**3.40**
**Interaction**	**125.23**	**6**	**20.87**	**3.77**	***0.00877***	**2.51**
Within	133.01	24	5.54			
Total	2864.80	35				

Table S11 – Statistic appraisal of impact of phytase (wheat) activity on zinc bioaccessibility Tukey's multiple comparisons test within zinc sources following ANOVA: Two Factor with Replication (n=3). (If in grey, not statistically significant; if in **Bold** $p < 0.05$ and if in ***bold italics***, $p < 0.01$).

Tukey's multiple comparisons test	Mean diff.	95.00% CI of diff.	Below threshold?	Summary	Adjusted P Value
		$ZnSO_4$			
(i) vs. (ii)	***13.39***	***8.09 to 18.69***	***Yes***	****	***<0.0001***
(i) vs. (iii)	***14.28***	***8.98 to 19.58***	***Yes***	****	***<0.0001***
(i) vs. (iv)	***10.22***	***4.92 to 15.52***	***Yes***	***	***0.0001***
(ii) vs. (iii)	0.8893	-4.413 to 6.19	No	ns	0.9665
(ii) vs. (iv)	-3.167	-8.47 to 2.14	No	ns	0.3723
(iii) vs. (iv)	-4.056	-9.36 to 1.25	No	ns	0.1785
		Zn bisglycinate			
(i) vs. (ii)	***17.67***	***12.37 to 22.97***	***Yes***	****	***<0.0001***
(i) vs. (iii)	***11.89***	***6.588 to 17.19***	***Yes***	****	***<0.0001***
(i) vs. (iv)	***17.05***	***11.75 to 22.36***	***Yes***	****	***<0.0001***
**(ii) vs. (iii)**	**-5.778**	**-11.08 to -0.48**	**Yes**	*	**0.0291**
(ii) vs. (iv)	-0.6142	-5.92 to 4.69	No	ns	0.9884
(iii) vs. (iv)	5.163	-0.13 to 10.47	No	ns	0.0583
		Zn proteinate			
(i) vs. (ii)	***20.58***	***15.28 to 25.88***	***Yes***	****	***<0.0001***
(i) vs. (iii)	***11.54***	***6.234 to 16.84***	***Yes***	****	***<0.0001***
(i) vs. (iv)	***15.08***	***9.774 to 20.38***	***Yes***	****	***<0.0001***
(ii) vs. (iii)	***-9.042***	***-14.34 to -3.74***	***Yes***	***	***0.0005***
**(ii) vs. (iv)**	**-5.502**	**-10.80 to -0.12**	**Yes**	*	**0.04**
(iii) vs. (iv)	3.54	-1.763 to 8.84	No	ns	0.2793

Table S12 – Statistic appraisal of impact of phytase (wheat) activity on zinc bioaccessibility Tukey's multiple comparisons test between zinc sources following ANOVA: Two Factor with Replication (n=3). (If in grey, not statistically significant; if in Bold $p < 0.05$ and if in bold italics, $p < 0.01$).

Tukey's multiple comparisons test	Mean diff.	95.00% CI of diff.	Below threshold?	Summary	Adjusted P Value
(i)					
**$ZnSO_4$ vs. Zn bisglycinate**	**-5.046**	**-9.846 to -0.2458**	**Yes**	*****	**0.0381**
$ZnSO_4$ vs. Zn proteinate	***-15***	***-19.80 to -10.20***	***Yes***	**********	***<0.0001***
Zn bisglycinate vs. Zn proteinate	***-9.957***	***-14.76 to -5.157***	***Yes***	**********	***<0.0001***
(ii)					
$ZnSO_4$ vs. Zn bisglycinate	-0.7661	-5.566 to 4.034	No	ns	0.9165
$ZnSO_4$ vs. Zn proteinate	***-7.813***	***-12.61 to -3.012***	***Yes***	********	***0.0013***
Zn bisglycinate vs. Zn proteinate	***-7.047***	***-11.85 to -2.246***	***Yes***	********	***0.0034***
(iii)					
**$ZnSO_4$ vs. Zn bisglycinate**	**-7.433**	**-12.23 to -2.633**	**Yes**	******	**0.0021**
$ZnSO_4$ vs. Zn proteinate	***-17.74***	***-22.54 to -12.94***	***Yes***	**********	***<0.0001***
Zn bisglycinate vs. Zn proteinate	***-10.31***	***-15.11 to -5.511***	***Yes***	**********	***<0.0001***
(iv)					
$ZnSO_4$ vs. Zn bisglycinate	1.787	-3.014 to 6.587	No	ns	0.6274
$ZnSO_4$ vs. Zn proteinate	***-10.15***	***-14.95 to -5.348***	***Yes***	**********	***<0.0001***
Zn bisglycinate vs. Zn proteinate	***-11.93***	***-16.73 to -7.134***	***Yes***	**********	***<0.0001***

Table S13 - Student's t-test with unequal variance and a two-tailed distribution (n=3). Statistic appraisal of zinc uptake of different zinc sources at 100 μM: in **A**. IPEC-J2 and **B.** Caco-2 cells (If in Bold $p < 0.05$ and if in bold italics, < 0.01).

A.

phytic acid: zinc	$ZnSO_4$	$ZnSO_4$	Zn bisglycinate
molar ratio	Zn bisglycinate	Zn proteinate	Zn proteinate
0:100	***0.008***	***0.0006***	0.22
10:100	**0.03**	0.11	0.57

B.

phytic acid: zinc	$ZnSO_4$	$ZnSO_4$	Zn bisglycinate
molar ratio	Zn bisglycinate	Zn proteinate	Zn proteinate
0:100	0.232	0.253	0.915
10:100	0.085	0.236	0.236

Table S14 - ANOVA: Two factor with replicates (n=3). Statistic appraisal of zinc uptake of different zinc sources at 100 µM in **A**. IPEC-J2 and **B.** Caco-2 cells (If in grey, not statistically significant; if in **Bold** $p < 0.05$ and if in ***bold italics,*** $p < 0.01$)

A.

Anova: Two-Factor With Replication					
SUMMARY	Digested control	$ZnSO_4$	Zn bisglycinate	Zn proteinate	Total
0: 100					
Count	3	3	3	3	12
Sum	20.30	93.33	132.57	141.93	388.13
Average	6.77	31.11	44.19	47.31	32.34
Variance	2.29	3.05	8.78	4.61	281.57
10: 100					
Count	3	3	3	3	12
Sum	17.45	97.02	135.53	126.70	376.70
Average	5.82	32.34	45.18	42.23	31.39
Variance	5.19	26.62	25.28	41.91	280.51
Total					
Count	6	6	6	6	
Sum	37.75	190.35	268.10	268.62	
Average	6.29	31.72	44.68	44.77	
Variance	3.26	12.32	13.92	26.33	

ANOVA						
Source of Variation	*SS*	*df*	*MS*	*F*	*P-value*	*F crit*
Sample	5.44	1	5.44	0.37	0.5518481	4.49
Columns	5909.16	3	1969.72	**133.86**	**1.524E-11**	3.24
Interaction	38.31	3	12.77	0.87	0.4781005	3.24
Within	235.43	16	14.71			
Total	6188.33	23				

B.

Anova: Two-Factor With Replication					
SUMMARY	Digested control	$ZnSO_4$	Zn bisglycinate	Zn proteinate	Total
0:100					
Count	3	3	3	3	12
Sum	11.46	56.23	87.40	84.46	239.55
Average	3.82	18.74	29.13	28.15	19.96
Variance	2.66	14.39	116.47	107.94	156.62
10:100					
Count	3	3	3	3	12
Sum	12.28	57.25	89.95	72.68	232.16
Average	4.09	19.08	29.98	24.23	19.35
Variance	2.03	27.26	39.66	11.35	115.42
Total					
Count	6	6	6	6	
Sum	23.74	113.48	177.35	157.14	
Average	3.96	18.91	29.56	26.19	
Variance	1.90	16.69	62.67	52.34	

ANOVA						
Source of Variation	*SS*	*df*	*MS*	*F*	*P-value*	*F crit*
Sample	2.27	1	2.27	0.06	0.8150824	4.49
Columns	2326.77	3	775.59	**19.28**	**1.456E-05**	3.24
Interaction	22.21	3	7.40	0.18	0.9056423	3.24
Within	643.52	16	40.22			
Total	2994.78	23				

Table S15 - ANOVA: Two factor with replicates (n=3). Statistical appraisal of zinc uptake of different zinc sources at 100 μM in **A**. IPEC-J2 and **B.** Caco-2 cells (If in grey, not statistically significant; if in **Bold** $p < 0.05$ and if in ***bold italics,*** $p < 0.01$).

A.

Tukey's multiple comparisons test	Mean diff.	95.00% CI of diff.	Below threshold?	Summary	Adjusted P Value
**0:100**					
**Digested control vs. $ZnSO_4$**	**-24**	**-33 to -15**	**Yes**	********	**<0.0001**
**Digested control vs. Zn bisglycinate**	**-37**	**-46 to -28**	**Yes**	********	**<0.0001**
**Digested control vs. Zn proteinate**	**-41**	**-50 to -32**	**Yes**	********	**<0.0001**
**$ZnSO_4$ vs. Zn bisglycinate**	**-13**	**-22 to -4.1**	**Yes**	******	**0.0036**
**ZnSO4 vs. Zn proteinate**	**-16**	**-25 to -7.2**	**Yes**	*******	**0.0005**
Zn bisglycinate vs. Zn proteinate	-3.1	-12 to 5.8	No	ns	0.754
**10:100**					
**Digested control vs. $ZnSO_4$**	**-27**	**-35 to -18**	**Yes**	********	**<0.0001**
**Digested control vs. Zn bisglycinate**	**-39**	**-48 to -30**	**Yes**	********	**<0.0001**
**Digested control vs. Zn proteinate**	**-36**	**-45 to -27**	**Yes**	********	**<0.0001**
**$ZnSO_4$ vs. Zn bisglycinate**	**-13**	**-22 to -3.9**	**Yes**	******	**0.0042**
**$ZnSO_4$ vs. Zn proteinate**	**-9.9**	**-19 to -0.93**	**Yes**	*****	**0.028**
Zn bisglycinate vs. Zn proteinate	2.9	-6.0 to 12	No	ns	0.784

**Digested control**					
0:100 vs. 10:100	0.95	-5.7 to 7.6	No	ns	0.7654
**$ZnSO_4$**					
0:100 vs. 10:100	-1.2	-7.9 to 5.4	No	ns	0.6992
**Zn bisglycinate**					
0:100 vs. 10:100	-0.99	-7.6 to 5.7	No	ns	0.7567
**Zn proteinate**					
0:100 vs. 10:100	5.1	-1.6 to 12	No	ns	0.1246

B.

Tukey's multiple comparisons test	Mean diff	95.00% CI of diff	Below threshold?	Summary	Adjusted P Value
0:100					
Digested control vs. $ZnSO_4$	*-15*	*-30 to -0.11*	*Yes*	*	*0.048*
Digested control vs. Zn bisglycinate	*-25*	*-40 to -11*	*Yes*	***	*0.0009*
Digested control vs. Zn proteinate	*-24*	*-39 to -9.5*	*Yes*	**	*0.0012*
$ZnSO_4$ vs. Zn bisglycinate	-10	-25 to 4.4	No	ns	0.2265
$ZnSO_4$ vs. Zn proteinate	-9.4	-24 to 5.4	No	ns	0.3015
Zn bisglycinate vs. Zn proteinate	0.98	-14 to 16	No	ns	0.9975
10:100					
Digested control vs. $ZnSO_4$	*-15*	*-30 to -0.18*	*Yes*	*	*0.0468*
Digested control vs. Zn bisglycinate	*-26*	*-41 to -11*	*Yes*	***	*0.0007*
Digested control vs. Zn proteinate	*-20*	*-35 to -5.3*	*Yes*	**	*0.0064*
ZnSO4 vs. Zn bisglycinate	-11	-26 to 3.9	No	ns	0.1936
ZnSO4 vs. Zn proteinate	-5.1	-20 to 9.7	No	ns	0.7553
Zn bisglycinate vs. Zn proteinate	5.8	-9.1 to 21	No	ns	0.6879

**Digested control**					
0:100 vs. 10:100	-0.27	-11 to 11	No	ns	0.9585
**$ZnSO_4$**					
0:100 vs. 10:100	-0.34	-11 to 11	No	ns	0.9483
**Zn bisglycinate**					
0:100 vs. 10:100	-0.85	-12 to 10	No	ns	0.8719
**Zn proteinate**					
0:100 vs. 10:100	3.9	-7.1 to 15	No	ns	0.4594

Table S16 - Statistic appraisal of zinc uptake of different zinc sources: p-values of statistical difference in uptake of zinc at concentrations reflective of recovery following *in vitro* digestion in **A**. IPEC-J2 and **B.** Caco-2 cells (If in **Bold** $p < 0.05$ and if in ***bold italics,*** $p < 0.01$).

A.

**phytic acid: zinc**	$ZnSO_4$	$ZnSO_4$	Zn bisglycinate
**molar ratio**	Zn bisglycinate	Zn proteinate	Zn proteinate
0:100	***0.005***	**0.04**	0.124
10:100	0.559	0.09	***0.0042***

B.

**phytic acid: zinc**	$ZnSO_4$	$ZnSO_4$	Zn bisglycinate
**molar ratio**	Zn bisglycinate	Zn proteinate	Zn proteinate
0:100	0.189	***0.012***	***0.033***
10:100	0.441	***0.026***	***0.028***

Table S17 - ANOVA: Two factor with replicates (n=3). Statistic appraisal of zinc uptake of different zinc sources at concentrations reflective of recovery following *in vitro* digestion in **A**. IPEC-J2 and **B.** Caco-2 cells (If in grey, not statistically significant; if in **Bold** $p < 0.05$ and if in ***bold italics,*** $p < 0.01$).

A.

Anova: Two-Factor With Replication					
SUMMARY	Digested control	$ZnSO_4$	Zn bisglycinate	Zn proteinate	Total
0:100					
Count	3	3	3	3	12
Sum	21.38	29.43	67.60	114.64	233.05
Average	7.13	9.81	22.53	38.21	19.42
Variance	2.78	1.59	6.88	119.26	189.10
10:100					
Count	3	3	3	3	12
Sum	7.81	18.81	16.39	25.21	68.23
Average	2.60	6.27	5.46	8.40	5.69
Variance	0.33	1.63	0.38	0.14	5.16
Total					
Count	6	6	6	6	
Sum	29.19	48.24	83.99	139.85	
Average	4.87	8.04	14.00	23.31	
Variance	7.39	5.04	90.32	314.36	

ANOVA						
Source of Variation	*SS*	*df*	*MS*	*F*	*P-value*	*F crit*
Sample	1132.01	1	1132.01	**68.10**	***3.6993E-07***	4.49
Columns	1183.40	3	394.47	**23.73**	***3.9277E-06***	3.24
Interaction	687.54	3	229.18	**13.79**	***0.0001061***	3.24
Within	265.98	16	16.62			
Total	3268.93278	23				

B.

Anova: Two-Factor With Replication

SUMMARY	Digested control	$ZnSO_4$	Zn bisglycinate	Zn proteinate	Total
0:100					
Count	3	3	3	3	12
Sum	15.22	23.24	33.14	52.50	124.11
Average	5.07	7.75	11.05	17.50	10.34
Variance	0.97	7.89	4.83	6.93	27.27
10:100					
Count	3	3	3	3	12
Sum	15.69	22.57	25.15	33.56	96.98
Average	5.23	7.52	8.38	11.19	8.08
Variance	0.40	1.99	0.99	1.10	5.77
Total					
Count	6	6	6	6	
Sum	30.91	45.81	58.29	86.07	
Average	5.15	7.64	9.72	14.34	
Variance	0.56	3.97	4.45	15.17	

ANOVA

Source of Variation	*SS*	*df*	*MS*	*F*	*P-value*	*F crit*
Sample	30.67	1	30.67	9.77	***0.00651***	4.49
Columns	273.38	3	91.13	29.04	***1.0418E-06***	3.24
Interaction	39.86	3	13.29	4.23	**0.02**	3.24
Within	50.21	16	3.14			
Total	394.13	23				

Table S18 – Tukey's test following ANOVA: Two factor with replicates (n=3). Statistic appraisal of zinc uptake of different zinc sources at concentrations reflective of recovery following *in vitro* digestion in **A**. IPEC-J2 and **B.** Caco-2 cells (If in grey, not statistically significant; if in **Bold** $p < 0.05$ and if in ***bold italics,*** $p < 0.01$).

A.

Tukey's multiple comparisons test	Mean diff.	95.00% CI of diff.	Below threshold?	Summary	Adjusted P Value
0:100					
Digested control vs. $ZnSO_4$	-2.7	-12 to 6.8	No	ns	0.8509
Digested control vs. Zn bisglycinate	***-15***	***-25 to -5.9***	***Yes***	**	***0.0014***
Digested control vs. Zn proteinate	***-31***	***-41 to -22***	***Yes***	****	***<0.0001***
$ZnSO_4$ vs. Zn bisglycinate	***-13***	***-22 to -3.2***	***Yes***	**	***0.0074***
$ZnSO_4$ vs. Zn proteinate	***-28***	***-38 to -19***	***Yes***	****	***<0.0001***
Zn bisglycinate vs. Zn proteinate	***-16***	***-25 to -6.2***	***Yes***	**	***0.0012***
10:100					
Digested control vs. $ZnSO_4$	-3.7	-13 to 5.9	No	ns	0.6936
Digested control vs. Zn bisglycinate	-2.9	-12 to 6.7	No	ns	0.8255
Digested control vs. Zn proteinate	-5.8	-15 to 3.7	No	ns	0.3355
$ZnSO_4$ vs. Zn bisglycinate	0.81	-8.7 to 10	No	ns	0.9948
$ZnSO_4$ vs. Zn proteinate	-2.1	-12 to 7.4	No	ns	0.9173
Zn bisglycinate vs. Zn proteinate	-2.9	-12 to 6.6	No	ns	0.8135

**Digested control**					
0:100 vs. 10:100	4.5	-2.5 to 12	No	ns	0.193
**$ZnSO_4$**					
0:100 vs. 10:100	3.5	-3.5 to 11	No	ns	0.3037
**Zn bisglycinate**					
**0:100 vs. 10:100**	**17**	**10 to 24**	**Yes**	***	**0.0001**
**Zn proteinate**					
**0:100 vs. 10:100**	**30**	**23 to 37**	**Yes**	****	**<0.0001**

B.

Tukey's multiple comparisons test	Mean diff.	95.00% CI of diff.	Below threshold?	Summary	Adjusted P Value
**0:100**					
Digested control vs. $ZnSO_4$	-2.7	-6.8 to 1.5	No	ns	0.288
**Digested control vs. Zn bisglycinate**	**-6**	**-10 to -1.8**	**Yes**	**	**0.0039**
**Digested control vs. Zn proteinate**	**-12**	**-17 to -8.3**	**Yes**	****	**<0.0001**
$ZnSO_4$ vs. Zn bisglycinate	-3.3	-7.4 to 0.84	No	ns	0.1443
**$ZnSO_4$ vs. Zn proteinate**	**-9.8**	**-14 to -5.6**	**Yes**	****	**<0.0001**
**Zn bisglycinate vs. Zn proteinate**	**-6.5**	**-11 to -2.3**	**Yes**	**	**0.002**
**10:100**					
Digested control vs. $ZnSO_4$	-2.3	-6.4 to 1.8	No	ns	0.4139
Digested control vs. Zn bisglycinate	-3.2	-7.3 to 0.98	No	ns	0.1708
**Digested control vs. Zn proteinate**	**-6**	**-10 to -1.8**	**Yes**	**	**0.004**
$ZnSO_4$ vs. Zn bisglycinate	-0.86	-5.0 to 3.3	No	ns	0.932
$ZnSO_4$ vs. Zn proteinate	-3.7	-7.8 to 0.47	No	ns	0.0923
Zn bisglycinate vs. Zn proteinate	-2.8	-6.9 to 1.3	No	ns	0.2519
**Digested control**					
0:100 vs. 10:100	-0.16	-3.2 to 2.9	No	ns	0.9152
**$ZnSO_4$**					
0:100 vs. 10:100	0.22	-2.8 to 3.3	No	ns	0.8787
**Zn bisglycinate**					
0:100 vs. 10:100	2.7	-0.40 to 5.7	No	ns	0.0843
**Zn proteinate**					
**0:100 vs. 10:100**	**6.3**	**3.2 to 9.4**	**Yes**	***	**0.0005**
